# Relationship between Functional Profile of HIV-1 Specific CD8 T Cells and Epitope Variability with the Selection of Escape Mutants in Acute HIV-1 Infection

**DOI:** 10.1371/journal.ppat.1001273

**Published:** 2011-02-10

**Authors:** Guido Ferrari, Bette Korber, Nilu Goonetilleke, Michael K. P. Liu, Emma L. Turnbull, Jesus F. Salazar-Gonzalez, Natalie Hawkins, Steve Self, Sydeaka Watson, Michael R. Betts, Cynthia Gay, Kara McGhee, Pierre Pellegrino, Ian Williams, Georgia D. Tomaras, Barton F. Haynes, Clive M. Gray, Persephone Borrow, Mario Roederer, Andrew J. McMichael, Kent J. Weinhold

**Affiliations:** 1 Department of Surgery, Duke University Medical Center, Durham, North Carolina, United States of America; 2 Los Alamos National Laboratory, Theoretical Division, Los Alamos, New Mexico, United States of America; 3 University of Oxford, Weatherall Institute of Molecular Medicine, Oxford, United Kingdom; 4 Nuffield Department of Clinical Medicine, University of Oxford, The Jenner Institute, Newbury, United Kingdom; 5 Department of Microbiology, University of Alabama at Birmingham, Birmingham, Alabama, United States of America; 6 VID Fred Hutchinson Cancer Research Center, Seattle, Washington, United States of America; 7 Departmental of Statistical Sciences, Baylor University, Waco, Texas, United States of America; 8 Department of Microbiology, University of Pennsylvania School of Medicine, Philadelphia, Pennsylvania, United States of America; 9 Department of Medicine, University of North Carolina at Chapel Hill, Chapel Hill, North Carolina, United States of America; 10 Department of Medicine, Duke University Medical Center, Durham, North Carolina, United States of America; 11 Centre for Sexual Health & HIV Research, Mortimer Market Centre, London, United Kingdom; 12 Department of Immunology, Duke University Medical Center, Durham, North Carolina, United States of America; 13 AIDS Research Unit, National Institute for Communicable Diseases, Johannesburg, South Africa; 14 Vaccine Research Center, National Institute of Health, Bethesda, Maryland, United States of America; Harvard University, United States of America

## Abstract

In the present study, we analyzed the functional profile of CD8^+^ T-cell responses directed against autologous transmitted/founder HIV-1 isolates during acute and early infection, and examined whether multifunctionality is required for selection of virus escape mutations. Seven anti-retroviral therapy-naïve subjects were studied in detail between 1 and 87 weeks following onset of symptoms of acute HIV-1 infection. Synthetic peptides representing the autologous transmitted/founder HIV-1 sequences were used in multiparameter flow cytometry assays to determine the functionality of HIV-1-specific CD8^+^ T memory cells. In all seven patients, the earliest T cell responses were predominantly oligofunctional, although the relative contribution of multifunctional cell responses increased significantly with time from infection. Interestingly, only the magnitude of the total and not of the poly-functional T-cell responses was significantly associated with the selection of escape mutants. However, the high contribution of MIP-1β-producing CD8^+^ T-cells to the total response suggests that mechanisms not limited to cytotoxicity could be exerting immune pressure during acute infection. Lastly, we show that epitope entropy, reflecting the capacity of the epitope to tolerate mutational change and defined as the diversity of epitope sequences at the population level, was also correlated with rate of emergence of escape mutants.

## Author Summary

An important role for the polyfunctional T-cell fraction of anti-HIV CD8 responses during chronic HIV infection has previously been suggested. This study characterized the role of polyfunctional T-cells directed against the transmitted/founder virus in the selection of viral escape mutants during acute HIV-1 infection within a unique cohort of individuals recruited within 3 weeks from the onset of symptoms at the time when the virus load was still declining. For the first time, the sequences of the transmitted/founder virus isolated from each patient were used. Interestingly, polyfunctionality was not found to be a pre-requisite for selection of escape mutations. A novel significant correlation is found between the order of appearance of escape mutations in different epitope sequences and both the magnitude of the CD8^+^ T-cell responses and the degree of entropy of the individual epitopes. A high proportion of the T-cells participating in the total response produced MIP-1β, suggesting that mechanisms not limited to the killing of infected cells might play a relevant role in early infection. This highlights the importance of measuring the quality of the CD8^+^ lymphocyte response and the sequence of the transmitted virus isolates to better understand the mechanisms of control of HIV replication during acute infection.

## Introduction

The development of vaccines capable of controlling infections by intracellular pathogens, including HIV-1, poses major challenges, since correlates of protection remain elusive [1], [2]. The initial observation that the appearance of HIV-specific CD8^+^ T cell responses is temporally associated with the resolution of peak viremia suggested that they may represent a critical component of initial protective immunity in humans [3], [4], [5]. Modeling based on the dynamics of the immune response and epitope escape data from very early in infection provides further support for the key role of CD8^+^ T cells responses in containing the virus during acute and early infection [6].

CD8^+^ T cell responses are undoubtedly able to place substantial pressure upon the virus, as indicated by the rapid appearance of escape mutations following HIV infection [7], [8], [9], [10]. In some cases, the appearance of these escape mutants is associated with the loss of virologic control, resulting in disease progression in HIV-1 [11], [12] and SIV infection [13], [14], [15]. However, CD8^+^ escape mutations do not always precede disease progression, and a number have been shown to result in a fitness cost that can lessen the replicative capacity of HIV [16], [17], [18], [19] and SIV [16], [20], [21].

Previous observations in chronic infection have shown that CD8^+^ T cell multifunctionality is associated, with slow HIV disease progression [22], [23]. CD8^+^ T cell responses exert significant immune pressure on HIV-1 in acute infection that results in rapid immune escape implying that they are important in early control of the virus [6], [24]. In the present report, we characterized the functional profile of CD8^+^ T cell responses directed against the transmitted/founder HIV-1 isolates arising during acute and early infection in order to determine the relationship between the functionality of epitope-specific CD8+ T cells and the selection of transmitted/founder virus escape mutants.

## Results

### Clinical parameters and course of viremia

**Figure 1:**
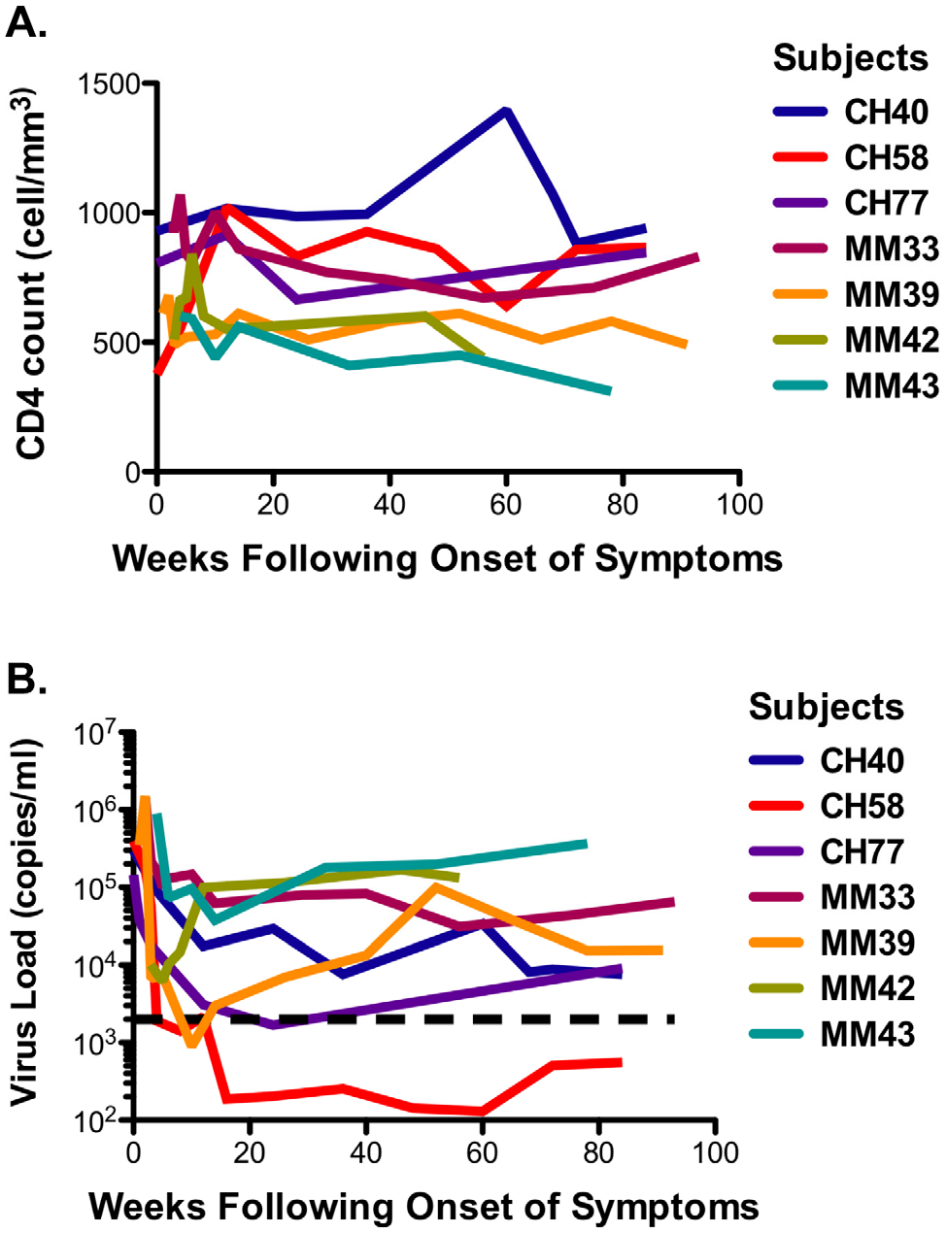
CD4 cell counts and virus load. **A**. The CD4 counts are reported in cell/mm^3^ at each time point following the onset of symptoms. **B**. The virus load in each subject is reported on a logarithmic scale as RNA copies/ml at each time point following the onset of the symptoms. The black dotted line represents the limits of 2,000 copies/ml used to identify controllers based on the CHAVI definitions of less than 2,000 copies/ml at 3 consecutive time points in 12 months.

The seven study participants were males between 23 and 56 years old at the time of diagnosis and all were infected as consequence of homosexual intercourse (MSM) (**Table 1**). The participants were enrolled within 3 weeks following onset of symptoms (FOSx) of acute HIV-1 infection. At the time of screening, the three patients enrolled in CHAVI 001 were classified as Fiebig stage 2, whereas the four patients enrolled at the Mortimer Market Center were stage 3. None elected to receive anti-retroviral therapy following diagnosis of acute HIV-1 infection and for the duration of the study. The analysis of the CD4 slopes (**Figure 1A**) and plasma virus load (pVL) (**Figure 1B**) were conducted according to previous publications [25] and revealed: 1) a sustained loss of CD4 cells in 4 patients (MM33, MM39, MM42, MM43), and 2) pVL set point at 40 weeks was above 2,000 copies/ml in all but patient CH058. In our study, a controller was classified as having a pVL of less than 2,000 RNA copies/ml at three consecutive time points during the first 12-month period (CH058, **Figure 1A**). Accordingly we identified one controller (CH058) and six non-controllers that each had greater than 2,000 copies/ml at multiple time points (**Figure 1A**).

**Table 1:** Patient demographic, CD4 slope, and plasma virus load set point, and class I HLA.

Patient ID/Class I HLA alleles	Gender	Age^a^	Transmission	Fiebig^b^	CD4 slope	pVL at Setpoint_wk40_ c
**CH040**	M	56	MSM	2	0.7727±2.095	7580
A*3101,*0201; B*4402,*4001; C*0302; C*0501						
**CH058**	M	23	MSM	2	2.733±2.336	199
A*0101,*2301; B*1402,*5701; Cw*0701 C*0802						
**CH077**	M	23	MSM	2	0.2687±1.994	5422
A*0205; B*5301,*5701; C*0401: C*1801						
**MM33**	M	36	MSM	3	−2.406±0.9509	57200
A*0201,*6801; B*0702,*4402; Cw*0501; Cw*0702						
**MM39**	M	37	MSM	3	−0.4987±0.5685	56350
A*0201,*0301; B*1501,*3501; Cw*09,*0401						
**MM42**	M	45	MSM	3	−3.593±1.984	151150
A*3201,*0201; B*0702,*3906; Cw*1502,*0702						
**MM43**	M	41	MSM	3	−3.254±0.8993	190050
A*0201; B*5501,*4001; Cw*10, *09						

a. Age at time of infection;

b. Fiebig stage at screening;

c. The data represent the average value of the plasma virus load (pVL) between week 36 and 56 in copies/ml.

### Functional profile of Gag-specific CD8^+^ memory T cell responses

**Figure 2:**
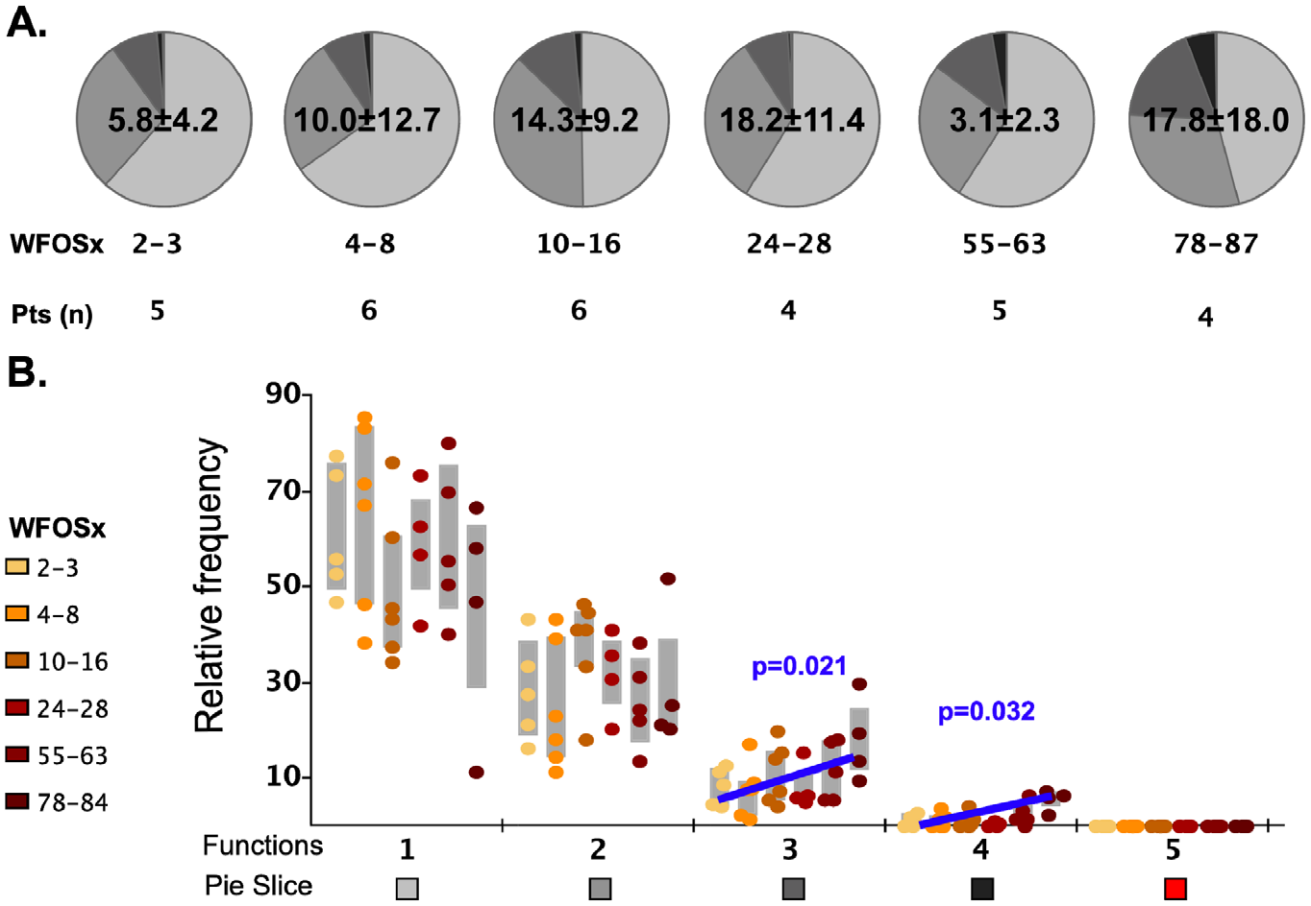
Functionality of Gag-specific responses. The total frequency of Gag-specific CD8+ memory T cells within the memory CD8+ T cell pool is reported within each pie as mean ± standard deviation. **A**) The pie charts represent the relative contributions of each functional family to the total response at each time point for the seven HIV-1 infected subjects. The color code for each number of functions is reported below the x-axis of the graph. The number of subjects studied at each time point is reported under each pie chart. **B**) In the graph each point represents one subject, and they are color coded according to the legend in the figure to represent the different weeks following onset of symptoms (WFOSx). Each grey box represents the inter-quartile distribution of the responses. The graphs represent the relative contribution to the total response made by each functional family at each time point. The trend line and p values for the statistically significant increase in subsets with 3 and 4 functions are also reported in the graph.

We initially analyzed the magnitude and kinetics of the total autologous Gag-specific CD8^+^ memory T cell response in each subject between 1 and 87 weeks (WFOSx) of acute HIV-1 infection along with associated multifunctional attributes of responding cells. Peripheral blood mononuclear cells (PBMC) cryopreserved at sequential time points FOSx were stimulated with a pool of overlapping peptides corresponding to the entire Gag sequence of the autologous transmitted/founder virus. The responses were evaluated by multiparameter flow cytometry, measuring the ability of CD8^+^ memory T cell subsets to degranulate (CD107a expression) and/or produce IFN-γ, IL-2, MIP-1β, and TNF-α (**Figure S1**). In 5 patients, Gag-specific CD8^+^ T cell responses were detected within 2–3 WFOSx and in all patients by 8 weeks WFOSx. The frequency of responding memory CD8^+^ T cells is reported within each pie chart (average ± standard deviation) in **Figure 2**. Analysis of the absolute magnitude of the responses did not reveal a reduction in the frequency of responses over time in each patient. The proportion of each responding population found to exhibit 1, 2, 3, 4 and 5 functions at each time point is presented in the pie charts (**Figure 2A**), while the relative frequencies of cells exhibiting each number of functions are reported in the graphs (**Figure 2B**). The analysis of the polyfunctional profiles among the seven patients indicated that Gag-specific CD8^+^ subsets mediating ≥3 functions contributed less than 20% of the total response in each subject at the two earliest time points (2–3 and 4–8 WFOSx) (**Figure 2A and 2B**). However there was some increase in the functionality of Gag-specific responses with time from infection. The comparison of contributions of each functional family to the overall response at different time points revealed that the families with 3 and 4 functions were significantly higher after 78 WFOSx compared to at earlier time points (p = 0.021 and 0.032, respectively, according to the LME model analysis), but remained below 20% of the total response in all but one instance. We also observed an absence (<0.005%) of the highly multifunctional IFN-γ^+^IL-2^+^MIP-1β^+^TNF-α^+^CD107^+^ subset at all time points tested. This was attributable to an absence of IL-2-producing CD8^+^ T cells (data not shown). The profile of the Gag-specific response in the controller CH058 was no different from those detected in the other six non-controller patients at any time points, and therefore it is not presented as segregated data.

### Polyclonal stimulation induces highly multifunctional CD8^+^ responses

**Figure 3:**
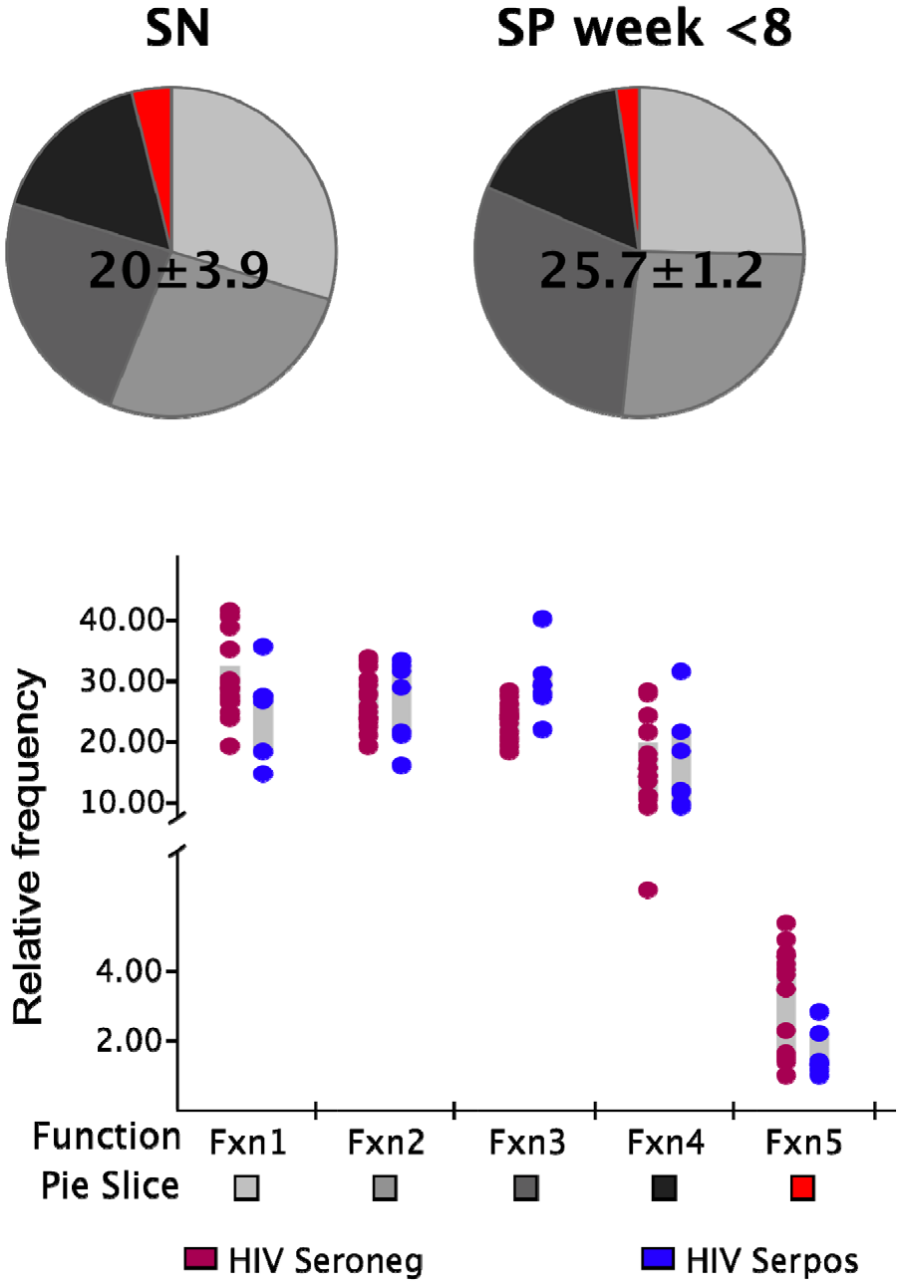
Functionality of SEB responses. The frequency of SEB-responsive CD8+ T cells (irrespective of their functional profile) within the total memory CD8+ T cell pool is shown above each pie. The pie charts represent the relative contribution of each functional family to the total response for the non-HIV-infected (SN; left pie charts) and infected donors (SP; right pie charts). The responses in the latter group represent those detected within the first 8 weeks post-onset of symptoms. The color code for each functional family is reported below the x-axis of the graph. Each dot represents one individual in the graph and color codes for the two groups are reported at the bottom of the figure (brown SN; blue SP). Each grey box represents the inter-quartile distribution of the responses. The graph represents the frequency of each functional family within the total SEB-responsive population for the subjects in each group.

We next determined whether this initial absence of highly multifunctional responses was due to a general functional impairment of the memory CD8^+^ T cell population. We stimulated PBMC from each HIV-infected and non-infected control subject with the superantigen *Staphylococcus aureus* enterotoxin B (SEB) in order to detect the presence of the different functional subsets in response to a polyclonal stimulus. The frequency of cells in the total CD8^+^ memory T cell population responding to SEB in the non-infected individuals was not significantly different (p>0.05) to that observed in the HIV-infected subjects within the first 8 WFOSx as reported in **Figure 3**. We detected every functional family including IFN-γ^+^IL-2^+^MIP-1β^+^TNF-α^+^CD107^+^ cells with a frequency greater than 0.05% in both HIV seronegative and seropositive individuals. This suggests that the limited contribution of cells with ≥3 functions may be confined to antigen-specific populations.

### Functional profiles of responses to mutating and non-mutating epitopes are similar

**Figure 4:**
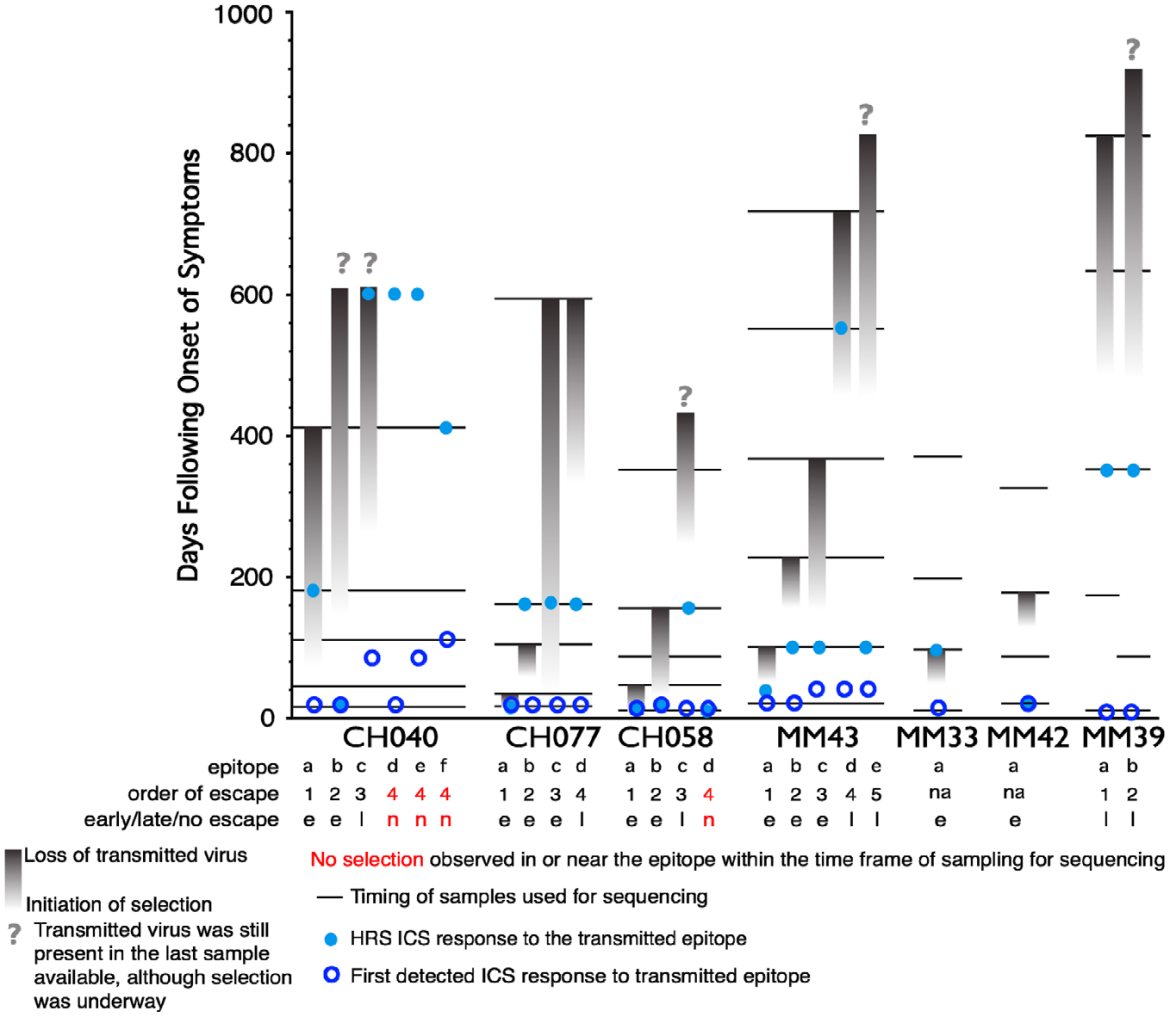
Timing of escape of each epitope relative to the first CD8+ T-cell responses. In studies of this kind, the timing of escape mutations and rate of loss of the transmitted form of epitope are based on limited temporal sampling of viral RNA from the plasma. Therefore, the time that immune escape was initiated can only be resolved to a period between the sample where only the transmitted epitope was present, and the sample where the first evidence of the accumulation of escape mutations appears. Similarly, the loss of the transmitted form can be prolonged or rapid, and in some cases was still ongoing at the last time point sampled. To illustrate and rank the order of escape while conveying the uncertainty of the rate and timing due to inevitable limitations in sampling, we used gradations of a grey bar, fading in during the period between the two samples when selection first is initiated, and ending at the sample where the transmitted form was no longer evident; the transmitted form diminishes through this period, and escape forms accumulate, indicated by the darkening gray. If the transmitted form was still present in the last available sample, we ended with a question mark to indicate selection was still ongoing. Four epitopes that elicited ICS responses that showed no evidence for escape during the course of the study are highlighted in red. Open blue circles indicate the timing of the sample where the first CD8+ T cell response was observed against the epitope, the light blue dots indicate time with the highest response to the epitope (HRS). The rank order of escape (reported for each epitope listed in **Table 2** within each patient by the lower case letter as ”epitope”) provides the values used for the correlation tests comparing order of escape to epitope entropy and the fraction of CD8+ T cell subtypes.

As the second aim of our study, we characterized the functional profile of CD8 responses directed against 23 individual epitopes previously mapped in the 7 subjects within HIV gene products not limited to Gag, as detailed in **Table 2**. The epitopic sequences are reported as those of the 18-mer peptides used to test the responses. 18-mer peptides were chosen for analysis of responses because we did not want to preclude characterization of responses that were not directed against previously described optimal epitopes (which was the case for 5 of the 23 epitopes reported in **Table 2**). In a side-by-side comparison, stimulation with representative 18-mers compared to optimal length peptides showed no differences in the functionality of the stimulated CD8^+^ T cells (data not presented), and we did not observe CD4^+^ T cell responses recognizing overlapping epitopes within the 18-mer peptides. The epitopes studied all elicited early T cell responses, previously mapped by ELISpot assay [6], [26], and could be detected by ICS within the first 6 WFOSx, except for epitopes c, e, and f in CH040, responses to which were first detected at week 12 (c and e) and 24 (f). As shown in **Figure 4**, we performed longitudinal sequence analysis of the viral quasispecies in each individual to reveal mutations within the epitope and/or 9 aa of the C- and N-flanking regions (all epitope variants are included in the **Figure S1**) that were either hypothesized or subsequently demonstrated to confer escape from the epitope-specific T cell response [6], [26]. Based on this definition, we detected the presence of escape mutations in twelve epitopes within 0–55 WFOSx and in seven additional epitopes between weeks 56 and 87. According to the time of detection of escape, the two groups are referred to as early (e-ME) or late (l-ME) mutating epitopes (ME) in **Table 2** **and** **Figure 4**, respectively. There was no evidence for mutation or escape in the other four epitopes within the time frame of this study; these are referred to as non-mutating epitopes (nME). The dynamics of appearance of escape and the loss of transmitted virus sequences are illustrated as the shaded gray bars above each epitope. According to the appearance of mutations, the epitopes are ranked within each patient both in **Table 2** and **Figure 4** (lower case letter in the “Epitope” column and row, respectively). The time of the earliest observed and the highest recorded frequency (HRF) of CD8 T cell responses for each epitope are also reported in **Figure 4**. Importantly, T cell responses were detected prior to the emergence of the epitope variants in 16 out of 17 escaping epitopes. The HRF occurred prior to the loss of transmitted virus in all epitopes but two, epitope “c” (Env 830–847) and “b” (Gag 236–235) in patients CH040 and CH077, respectively.

**Table 2:** Analysis of Entropy of sequences and epitope-specific functional responses based on the order of time of appearance of epitope mutations within each individual.

										Earliest Positive ICS i			
	HIV Antigen/aa position a	Sequence b	Allele restriction c	Week d	VL c/ml e	Epitope f	Order of escape	Mutation g	Entropy h	Total Freq	Fxn 1	Fxn 2-5	MIP-1β^+^
**Patient ID**							**within PID**						
**CH040**	Rev 49–66	QR**QIRSISERIL**STYLER	A*0201	4	89,156	a	1	e-ME	5.07	0.48	0.00	0.48	0.46
	Vif 113–129	DCFSES**AIRKAILGRI**VS	A*3101	4	89,156	b	2	e-ME	3.36	0.58	0.23	0.35	0.52
	Env 830–847	IEVVQRACRAILHIPRRI	undetermined	12	17,587	c	3	l-ME	3.31	0.28	0.09	0.19	0.24
	Env 665–682	LFSYH**RLRDLLLIV**TRIV	A*0201	4	89,156	d	4	nME	3	0.30	0.05	0.25	0.30
	Gag 481–498	**KELYPLASL**RSLFGNDPS	B*4001	12	17,587	e	4	nME	3.09	0.39	0.12	0.27	0.28
	Pol 824–841	VKTIHTDNGSNFTSTTVK	undetermined c	24	29,453	f	4	nME	1.43/3.08	0.39	0.06	0.33	0.34
**CH058**	Env 581–596	LAL**ERYLRDQQL**LGIW	B*1402/Cw*0702	2	181,262	a	1	e-ME	1.48	3.60	1.63	1.97	3.37
	Gag 236–253	IAGS**TSTLQEQIGW**MTSN	B*5701	2	181,262	b	2	e-ME	1.29	0.87	0.73	0.15	0.79
	Gag 140–157	GQMVHQA**ISPRTLNAW**VK	B*5701	4	1,908	c	3	l-ME	0.83	0.91	0.54	0.37	0.45
	Nef 113–130	WVY**HTQGYFPDW**QNYTPG	B*5701	2	181,262	d	4	nME	1.05	1.69	1.04	0.65	1.52
**CH077**	Env 350–368	HVVDKLRE**QFRNKTIVF**NH	Cw*0401	4	17,907	a	1	e-ME	4.99	0.71	0.60	0.11	0.59
	Gag 236–253	IAGS**TSTLQEQVGW**MTSN	B*5701	4	17,907	b	2	e-ME	1.29	0.42	0.21	0.21	0.32
	Env 334–351	SGEDWNKTLSHVVDKLRE	undetermined	4	17,907	c	3	e-ME	5.54	0.46	0.30	0.16	0.41
	Env 605–622	T**TTVPWNVSW**SNKSLNEI	B*5701	4	17,907	d	4	l-ME	2.69	0.31	0.27	0.04	0.19
**MM33**	Pol 81–98	DTGADDTVL**EEMNLPGRW**	B*4402	2	1,451,400	a	na	e-ME	2.82	0.51	0.43	0.08	0.52
**MM39**	Nef 65–82	EVGFPVRP**QVPLRPMTYK**	A*0301	1	350,600	a	na	l-ME	1.79	0.14	0.14	0.00	0.06
	Gag 17–34	EKI**RLRPGGKKK**YKLKHI	A*0301	1	350,600	b	na	l-ME	1.48	0.28	0.25	0.03	0.12
**MM42**	Nef 185–202	FDSRLAFHHIARELHPEY	A*0201	4	7,800	a	na	e-ME	2.43	0.65	0.57	0.08	0.56
**MM43**	Vif 113–130	DCFSESAIRGAILGHIVS	undetermined	4	898,100	a	1	e-ME	2.46	1.89	1.41	0.49	1.09
	Nef 177–194	EKEV**LEWRFDITL**AHHHR	A*0201	4	898,100	b	2	e-ME	4.61	1.27	0.75	0.52	1.10
	Env 6–20	NYQHLWRGGIMLLWRGIM	undetermined	6	73,300	c	2	e-ME	3.06	0.95	0.77	0.18	0.90
	Nef 89–106	FFL**KEKGGLEGL**IHSQKR	B*0140	6	73,300	d	4	l-ME	1.15	0.90	0.61	0.29	0.85
	Pol 633–650	ELQAIHL**ALQDSGLEV**NV	A*0201	6	73,300	e	4	l-ME	1.51	0.79	0.60	0.32	0.79
								**p = **	**0.007**	**0.03**	**0.11**	**0.07**	**0.006**

a.The position refers to the position in the HXB2 sequence.

b.Optimal defined or predicted epitopic sequences are reported in bold.

c. "Undetermined": no epitopes recognized within restricting alleles expressed by the patients have been previously reported.

d. Indicates time of the earliest frequency of response detected by ICS assay.

e. Virus load (VL) detected at the correspondent week of T cell response (d) is reported in copies/ml.

f. The letters indicate the ranking based on appearance of escape mutations or lack thereof. The same letter are used in Figure 5 to indicate the correspondent epitope.

g. Epitopes dysplaying mutations related to T cell responses and evidence for variation escape within 55 and 87 WFOSx are listed as early (e-ME) or late (l-ME), respectively; those without evidence for variation and escape are reported as nME. Escape mutant epitopes are listed in order of appearance within each subject.

h. The numbers reflect the Shannon entropy as indicated in the Methods section. The higher is the value, the higher is the likelihood for the region to allow for variability in the sequence.

i. Each column reports the frequency (Freq) of the population of interest as % of total memory CD8+ T cells.

We further analyzed the responses according to the profile of the functional families at the earliest detectable time point, and at the time of HRF of the response. At the level of group analysis within the first 24 WFOSx (**Figure 5A**), we observed greater representation of the monofunctional subset in the responses against the early and late mutating epitopes than those against the non-mutating epitopes, whereas cells mediating 2 and 3 functions were relatively more abundant in the non-mutating-epitope-specific responses (>30%). These profiles changed slightly at the time of HRF, which for 12 out 23 (52%) epitope-specific responses was after 24 WFOSx. There was some overall increase in functionality with the 2- and 3-functional subsets contributing more to the responses for each group of epitopes, in line with the data reported in **Figure 2**. Interestingly, the 4-functional subset contributed more to responses to the non-mutating epitopes (**Figure 5B**). We confirmed that the IFN-γ^+^IL-2^+^MIP-1β^+^TNF-α^+^CD107^+^ subset made a negligible contribution to the overall response, with frequencies <0.005% at all time points analyzed.

**Figure 5:**
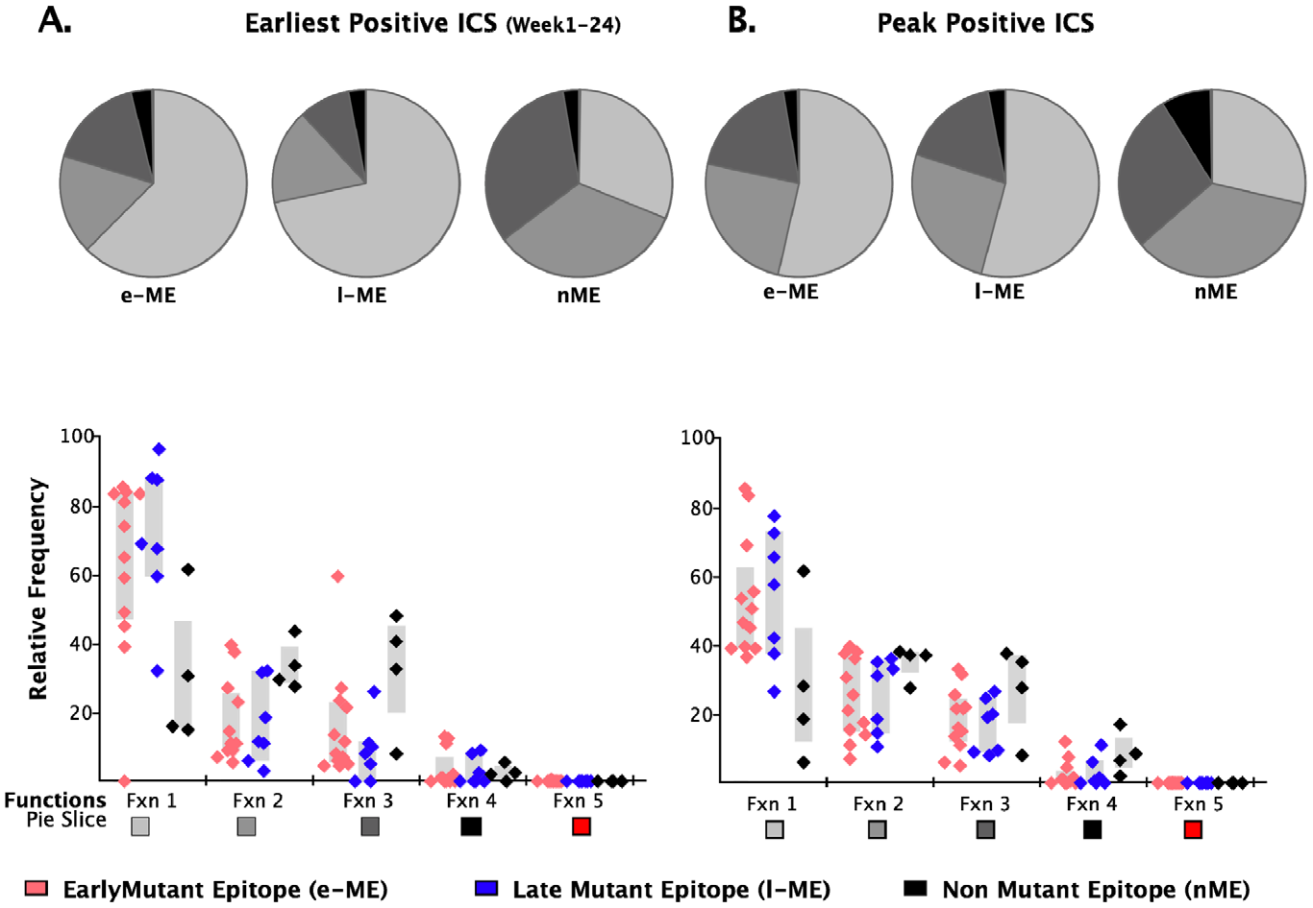
Analysis of functional profile of responses to mutating and non-mutating epitopes. The relative contributions of each functional family to the total CD8 T cell response are reported within each pie chart for epitopes that mutated within 55 (early-mutating: e-ME) and 56–87 WFOSx (late-mutating: l-ME) and for those that did not mutate (non-mutating: nME) epitopes. **A**) Profile of responses within 12 weeks FOSx. **B)** Profile of responses at the time of detection of the highest response by ICS (peak). The color code for each functional family is reported below the x-axis of the graph. In the graph each dot represents a single epitope. The color code for each group of epitopes is reported at the bottom of the figure. The grey bar represents the inter-quartile distribution of relative frequency of each functional family for the e-ME-, l-ME, and nME responses.

### Epitope sequence entropy and total frequency of functional CD8^+^ T cell responses are both associated with the temporal appearance of escape mutants

Given no clear correlation between functionality and virus escape, we hypothesized that rapid selection for CD8^+^ T cell escape mutants might be a function of both the intrinsic ability of the targeted epitope to tolerate change as well as the patient's immune pressure exerted by the epitope-specific CD8^+^ T cell responses whether detected at the earliest or HRF time point. To provide a relative measure of how readily the epitopes included in this study tolerate change, we calculated the Shannon entropy of each epitope based the full collection of B clade sequences in the Los Alamos database. Highly conserved epitopes will have a low entropy score, and variable epitopes that frequently are altered and clearly tolerate many patterns of substitution at the population level will have a high entropy (see Methods for details). We indicate in **Figure 4** when the first evidence for selection and the last traces of the transmitted epitope were observed. In some cases the population completely shifted from transmitted to escape variants between 2 sampling time points. In other cases selection manifested gradually, with escape mutations accruing slowly over many months. In 4 cases, escape mutations in recognized epitopes did not arise within the time frame of the study. We ranked the order of escape for each epitope within each subject based on the temporal appearance of mutations (**Table 2** and **Figure 4**). By using a Spearman's ρ statistic as described in the methods to assess correlations, we observed a significant association of the temporal appearance of mutation with both the entropy of the epitope (p = 0.007) and the total frequency of CD8^+^ T cell responses detectable at the earliest time point (p = 0.03) as reported in **Table 2**.

### Relative contribution of T cells producing MIP-1β to the overall response is significantly greater for early-ME-specific responses

**Figure 6:**
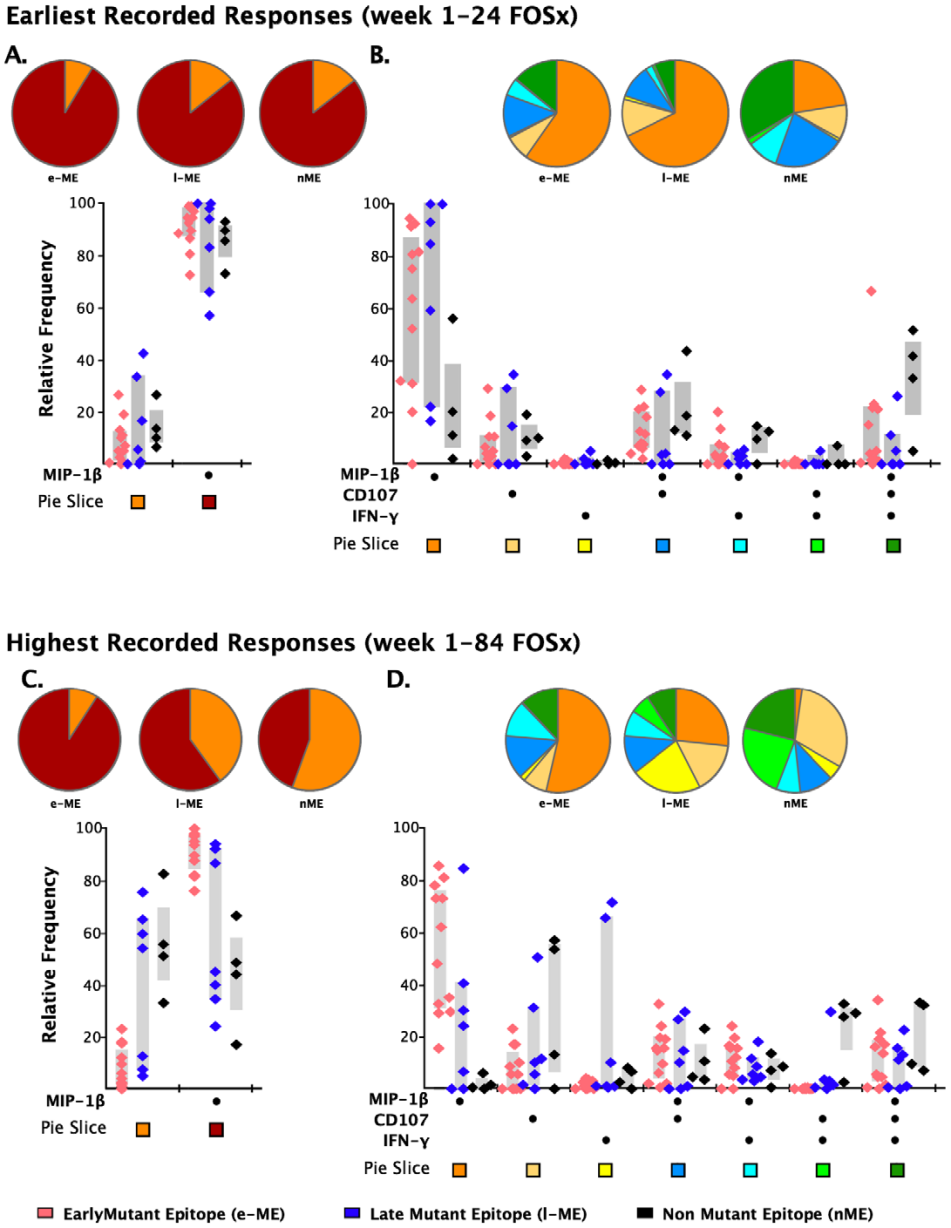
Analysis of individual functions and polyfunctional subsets. The analysis of the responses at the earliest (panels **A** and **B**) and highest recorded (panels **C** and **D**) time points are shown. The pie charts represent the relative contribution to the total response of cells exhibiting each functional parameter or combination of functional parameters. The color code for each parameter (pie slice) is reported below the x-axis of the graph. In the graph, each triangle represents the early (e-ME), late (l-ME), and non-mutating (nME) epitope-specific CD8+ T cell response detected at each time point by ICS. The color code for each group of epitopes is reported at the bottom of the figure. The grey bars represent the inter-quartile distribution of the relative frequency of each functional parameter. The dots under the x-axis represent the positive responses for each parameter. (**A and C**) Total MIP-1β^+^ responses are compared to the total responses detected as any combination of the other parameters excluding MIP-1β. (**B and D**) Analysis of the single or combined contribution of degranulation (CD107), IFN-γ and MIP-1β production is represented by the dots under the x-axis.

As analysis of the functional families at the individual patient level revealed an association of total responses and ability to select for escape mutations, we investigated whether this association may reside at the level of any single or combination of the five functional parameters. We noticed that CD8^+^IL2^+^ T cells did not contribute to any of the three groups of epitope-specific responses and that the majority of CD8^+^TNF-α^+^ were also CD8^+^IFN-γ^+^ T cells, therefore, we analyzed the remaining response subsets without regard to production of IL2 and TNF-α. We observed that the earliest recorded frequency of the CD8^+^ T cells producing MIP-1β^+^ alone and in any combination with any other parameter was also significantly associated with the appearance of escape responses within subjects (**Table 2**, p = 0.006). A complication in interpreting the role of MIP-1β^+^CD8^+^ T cells on immune pressure is that their frequency is highly correlated with the total ICS memory fraction at the earliest time point, making it difficult to resolve which parameter, the total response or MIP-1β^+^, is driving the pace of immune escape. The median relative contribution of total CD8^+^MIP-1β^+^ T cells was >80% to all ME-specific responses at early time points, as shown in **Figure 6A**, and declined with time for the l-ME and nME groups as shown in **Figure 6C**. We further analyzed whether degranulation or IFN-γ production were also associated with the significant predominant contribution of CD8^+^MIP-1β^+^ cells to responses to e-ME. As shown in **Figure 6B and 6D**, among the possible CD8^+^MIP-1β^+^ subsets with more than one function we could identify that both CD8^+^MIP-1β^+^CD107^+^ and CD8^+^MIP-1β^+^CD107^+^IFN-γ^+^ subsets contributed to the e-ME-specific responses but also made a similar contribution to the l-ME and nME responses. Moreover, segregate analysis of both total CD8^+^IFN-γ^+^ (p = 0.99) and total CD8^+^IFN-γ^+^ MIP-1β^+^ (p = 0.27) subsets did not reveal any significant association with the appearance of escape mutants. Thus, we observed that the MIP-1β-producing CD8^+^T cells represent the dominant proportion of cells detectable in the early stage of infection prior to virus escape, and that this was strongly associated with immune pressure resulting in selection of the early escape mutants.

## Discussion

The results of both the STEP and RV144 clinical trials suggest that CD8^+^ T cell responses alone might neither prevent HIV-1 infection nor lower virus set-point after infection [27], [28]. However, given the relatively weak and narrow T-lymphocyte responses were induced by the vaccine protocol used the in the STEP trial [29], and encouraging non-human primate vaccine studies [30], [31], [32] it is still important to continue this approach. Thus, features of HIV-specific CD8^+^ T cells that determine their *in vivo* efficacy need to be defined. It has been widely documented that CD8 T cell responses are elicited early following HIV-1 infection and that they are associated with control of viremia as well as with selecting for escape mutants that may impact virus fitness [33]. As their most important impact may take place during acute infection, we have focused our study on characterization of the functionality of HIV-specific CD8 T cell responses at this time, concentrating on epitope-specific responses which did or did not drive selection for escape mutations of the transmitted virus.

We evaluated CD8^+^ T cell responses using 18-mer peptides corresponding to the autologous transmitted/founder viral sequence of seven individuals recruited within 45 days from the onset of acute HIV-1 infection [6], [26]. We first studied the total response to the *gag* gene product, because of its importance in vaccine design, and then extended the study to epitopes that we had previously characterized for escape mutants because of their potential impact on virus set point [6], [26]. We observed that: 1) more than 80% of the epitope specific T cells in acute infection were oligofunctional; 2) MIP-1β-producing T cells were associated with epitopes that escaped; 3) transmitted/founder virus escape mutants in acute infection occur in epitopes that have high entropy at the population level.

Previously in chronically infected patients, multifunctionality of HIV-specific CD8^+^ T cell responses in the peripheral blood has been shown to be associated with long-term non-progression [22], [23], and it has been related to the CD4 count and inversely related to the virus load at the rectal mucosal level [34]. In our study, we characterized the earliest functional responses and observed that the early CD8 T cell response was predominantly mono- or bi-functional. The contribution of T cells with 3-5 functions was small but increased with time from infection and we cannot exclude that they become a major subset as the infection progresses, particularly in those who control the infection well, as previously described. Although we cannot exclude the possibility that the presence of early poly-functional CD8^+^T cell responses may predict control of viremia, the observation that the only controller in our cohort displayed a profile of functional responses similar to those who did not control virus replication would suggest otherwise. Our observations are largely in agreement with those made by Streeck and collaborators, who reported an increase in poly-functionality with the appearance of escape mutants and a subsequent decrease in functionality over time [35]. In fact, we likewise observed an increase in functionality of epitope-specific CD8^+^ T cell responses from the early to the HRF time point, although, we showed that only the total CD8^+^ T cell responses were significantly associated with the appearance of escape mutations. This could be related to the fact that, in our study, responses were detected in most cases before full escape occurred when virus load had not yet reached the virus set-point. The initially low level of multifunctionality might be related to high levels of virus replication that would in turn favor upregulation of inhibitory receptors such as PD1. Such upregulation would have a negative impact on the functionality of the CD8^+^ T cells – an impact that is rescued in chronically-infected individuals upon control of VL following initiation of therapy [36], [37]. By comparing our data to those obtained from individuals immunized with MVA to protect against smallpox virus infection [38], we also hypothesize that HIV-specific CD8 responses may be substantially delayed in acquiring multifunctionality because of impairments in CD4^+^ T cell populations, which are otherwise preserved and functional in MVA vaccine recipients. It is, therefore, possible that maturation of HIV-1 specific multifunctional CD8^+^ T cell responses might be affected by both the impact of high levels of virus replication and the early loss of CD4^+^ T helper function.

In the present study we also sought to characterize whether the functionality of the CD8^+^ T cell responses generated during acute/early HIV-1 infection may be related to the immune pressure exerted on the transmitted virus. For the 19 ME epitope-specific CD8^+^ T cell responses studied, the total magnitude of functional CD8^+^ T cell response at early time points was significantly associated with the early appearance of escape mutations, when responses were mainly oligofunctional. Multifunctional T cells were a minor component of the response. The observation of the overall impact of the magnitude of functional T cell responses on the appearance of escape mutations is in agreement with previous results obtained by the analysis of IFN-γ^+^CD8^+^ T cell responses, although, in this cohort of patients the IFN-γ^+^ responses alone did not show significant association with the appearance of escape mutants [26], [39]. Other characteristics of the T cell responses to e-ME may also have influenced the rate at which they were escaped. For example, e-ME may generally elicit clonal CD8^+^ T cell populations with higher avidity [40], [41], [42] and/or a better selection of a T cell receptor (TcR) repertoire, as suggested by both human and NHP studies [42], [43]. These questions are being addressed separately.

Further analysis of the proportion of epitope-specific T cells producing individual cytokines revealed for the first time that MIP-1β^+^-producing CD8^+^ T cells constituted greater than 80% of the cells participating in the early responses and, therefore, were associated with the appearance of escape mutants. The contribution of MIP-1β^+^-producing CD8^+^ T cells to the overall response declined with time for the l-ME and nME responses, suggesting that persistent antigenic stimulation might also be an important factor in selecting for clones whose functional profile is dominated more by IFN-γ^+^ production as part of the maturation process [44]. Among the CD8^+^MIP-1β^+^ responding cells, we observed that CD8^+^MIP-1β^+^CD107^+^ and CD8^+^MIP-1β^+^CD107^+^IFN-γ^+^ T cells were the two multifunctional subsets most highly contributing to the early responses. We have recently demonstrated that CD8^+^ T cell mediated antiviral function in virus controllers and vaccinees is associated with Clade B Env and Gag-specific MIP-1β and CD107a expression as monofunctional and dual-functional populations, suggesting the importance of both cytolytic and non-cytolytic mechanisms in control of HIV replication [45]. CD8^+^ T cells can suppress HIV-1 replication noncytolytically through the secretion of a number of soluble factors, including the B-chemokines, MIP1α, MIP1β and RANTES, which can potently inhibit CCR5 tropic virus infection [46]. We have found that the secretion of MIP-1α, MIP-1β, IP-10, MIG, IL-1, and interferon gamma correlated most strongly with soluble noncytolytic suppression [47]. Using the ability of histone hyperacetylation to alter the expression of immune-related genes, we identified that MIP-1α and IP-10 were also among the genes that were down-regulated by histone hyperacetylation; again supporting the idea that they contributed to soluble CD8^+^ T cell mediated suppression [47], [48]. Taken together, these observations suggest that T cells involved in selecting for escape mutants during acute HIV-1 infection may possess a combination of activities that include both killing and inhibitory activity. This possibility has also been suggested in the SIV model where loss of HIV-infected CD4^+^ T cells was not prevented by administration of antiretroviral therapy in CD8-depleted animals, suggesting that CD8^+^ T cell functions other than cytotoxic activity might contribute to early control of virus replication and recovery of CD4 counts [49], [50].

Neither the magnitude, the phenotype, nor the functionality of CD8^+^ T cell responses at time of HRF showed any association with the appearance of escape mutants. These lack of associations could be related to several factors: 1) our inability to detect the true peak of responses; 2) the fact that the maturation of responding cells may be impaired by high levels of virus replication; and 3) rapid appearance of escape mutants.

Our data indicate that high entropy at the population level is significantly associated with the appearance of escape within the first six months post-infection, extending observations made in previous studies of escape in acute infection [51], [52]. Epitopes that exhibited delayed escape despite an early response were associated with low entropy at the population level, hence higher fitness costs may be associated with change in these epitopes. Our finding is in line with results from earlier studies that have demonstrated the role of fitness costs of sequence changes in/around CD8+ T cell epitopes in constraining escape from epitope-specific responses [6], [16], [26]. Delayed escape was also associated with less vigorous CD8^+^ T cell responses as revealed by lower total response magnitudes assessed by ICS.

Overall, our data show that fully multifunctional (5+) Gag-specific CD8^+^ T cell responses are not detectable during the initial weeks following HIV-1 infection in the response to the transmitted virus. Moreover, the relative contribution of 3- and 4-multifunctional subsets to the total response is small, although it increases with time from initial infection. Importantly, the magnitude of epitope-specific CD8^+^ T cell responses including the frequency of epitope-specific MIP-1β -producing cells is associated with the order of appearance of escape mutants. The order in which different epitopes escaped CD8 responses is also associated with the population diversity (entropy) of the epitopes. These findings underscore the important role that the magnitude of the CD8^+^ T cell responses plays in exerting pressure on replication of transmitted/founder HIV-1 in the early stages of infection, and illustrate the importance of considering both epitope entropy and functional responses beyond the cytotoxic properties of epitope-specific CD8^+^ T cells for vaccine design.

## Methods

### Subjects

The seven individuals enrolled in this study were identified as acutely HIV-1-infected by the presence of detectable HIV-1 viral RNA in their plasma, but either negative or discordant HIV-1 serology at the screening visit. Three infected patients (CH040, CH058 and CH077) were enrolled in the CHAVI 001 study at the Duke University Medical Center and Chapel Hill UNC-Hospital (USA). Four additional patients (MM33, MM39, MM42 and MM43) were recruited at the Mortimer Market Center (London, UK). CD4 cell counts and viral loads were determined by local accredited clinical laboratories. The Fiebig stage was determined as previously reported [53]. All seven individuals remained HAART naïve for the duration of the study.

PBMC were isolated by standard density gradient centrifugation from anti-coagulated blood samples, within 8 hours of collection. PBMC were viably cryopreserved in fetal calf serum supplemented with 10% DMSO and stored in vapor phase liquid nitrogen.

PBMC samples were also obtained from 16 HIV-1 seronegative individuals as a control group.

Each participant enrolled in the study provided written informed consent in conjunction with protocols approved by the institutional review boards of Duke University Medical Center and the Mortimer Market Center for Sexual Health and HIV research.

### Sequence analysis of transmitted virus isolates

Sequences of the transmitted virus were derived from plasma obtained at either the screening or enrollment visit. RNA extraction, complementary DNA synthesis, and sequence analysis were performed as previously described [26], [54]. GeneBank accession numbers for sequences are as follows: HM586186-HM586209 correspond to 24 bulk sequences from MM33, MM39, MM2, and MM43; HM153284-HM153412 correspond to 129 SGA sequences from MM39 and MM43; and FJ495804-FJ496071, FJ496086-FJ496144, and FJ919955-FJ919967 include 340 SGA sequences derived from CH40, CH58, and CH77. Alternatively, longitudinal alignments from subjects CH40, CH58 and CH77 can be found at http://www.hiv.lanl.gov/content/sequence/HIV/USER_ALIGNMENTS/Salazar.html as previously published [54].

### Peptides

Eighteen amino acid peptides, overlapping by 10 amino acid residues, were synthesized based on the autologous transmitted HIV-1 sequences for each infected study subject as previously described [6], [26].

### T cell epitope mapping

The viral peptides recognized by the primary HIV-specific T cell response in each patient were identified by *ex vivo* interferon-gamma (IFN-γ) ELISpot assay as previously reported [6], [26]. Briefly, PBMC were thawed and, after overnight (CH040/058/077–2 hr resting) resting, stimulated with peptide pools at a final concentration of 2 µg/ml for 20–24 hr at 37°C in 5% CO_2_. For each assay, six negative control wells (no antigen) and triplicate wells of at least 1 positive control (PHA at 10 µg/ml; Sigma-Aldrich) were included. Each epitope-specific response identified from the screening was confirmed in a secondary assay, and, for some responses, by testing the recognition of shorter peptides A criteria of positivity of ≥50 Spot Forming Cells (SFCs)/10^6^ cells and >3x background was established.

### Multi-parameter intracellular cytokine staining assay

Cryopreserved PBMC were thawed and rested overnight. PBMC were stimulated with individual or pools of peptides representing the sequence of the autologous transmitted HIV-1 isolate. For all subjects, a polyclonal stimulation with the superantigen *Staphylococcus aureus* enterotoxin B (SEB) was also used as positive control. Each antibody was titered to determine the saturating concentration used for the final staining. The stimulations were conducted in the presence of 1 µg/ml anti-CD28 mAb (clone L293; BD), 1 µg/ml anti-CD49d mAb (clone L25; BD), anti-CD107a Alexa Fluor 680 (clone H4A3; BD), 5 µg/ml brefeldin A (Sigma-Aldrich) and 1 µg/ml of Golgi Stop (BD) for 5.5 h at 37°C in 5% CO2. After washing, the cells were surface stained with anti–CD4-Cy5.5-PE (clone M-T477; Biolegend), anti–CD8-QD705 (clone RPA-T8; InVitroGen), anti–CD27-Cy5-PE (clone M-T271, BD Bioscience), anti–CD57-QD565 (clone NK-1, AbD; conjugated in M. Betts Lab), and anti–CD45RO-PE–Texas Red (clone UCHL1, Beckman Coulter) for 20 min at room temperature. Admixtures of anti–CD14–Cascade blue (clone M5E2, Biolegend), anti–CD19–Cascade blue (clone HIB 19, Biolegend), and a vital dye (ViVid; InVitroGen) were also used to exclude monocytes/macrophages, B cells, and non-viable cells from the analysis.

PBMC were subsequently fixed and permeabilized with Cytofix/Cytoperm and Perm/Wash buffer (Pharmingen, San Diego, CA) for 20 minutes, washed twice, and stained with anti–CD3-Cy7-APC (clone SK7, BD Bioscience), anti–IFN-γ–FITC (B27), anti–IL-2–APC (clone MQ1-17H12), anti–TNF-α–Cy7-PE (clone MAb11), and anti–MIP-1β-PE (clone D21-1351; all obtained from BD) for 1 h at 4°C. After washing and fixation, samples were acquired on a custom made LSRII (BD Bioscience, San Jose, CA) within the next 24 hours. A minimum of 300,000 total viable events was acquired for each test.

### Gating strategy

Gates were set to include singlet events, live CD3^+^ cells, lymphocytes, and CD4^+^ and CD8^+^ subsets (Figure 1). From the total CD8^+^ population, the naive subset was identified as CD45RO^−^CD27^+^. This subset was excluded from the subsequent analysis, and only the memory population was included, indicated by the red gate in **Figure S1.I**. Within the memory population, the central memory CD45RO^+^CD27^+^ (CM; RO^+^27^+^), effector CD45RO^+^CD57^+^ (E; RO^+^57^+^), and the terminal effector CD45RO^−^CD57^+^ (TE; RO^−^57^+^) populations were identified.

Antigen-specific populations were identified within the memory population as single-function cells shown in the sequential single cytokine/chemokine/degranulation gates. Thirty-two poly-functional subsets were identified using a Boolean gating strategy that included both the functions and memory phenotype of the cells. Based on the initial 32 functional subsets, we calculated the frequency of the total CD8^+^ memory response as well as of the total frequency of families of subsets having the same numbers of functional parameters as shown in **Figure S1.II**.

### Data analysis

Data analysis was performed using FlowJo 8.8.4 software (TreeStar), Pestle for background subtraction, and SPICE for frequency analysis. Pestle and SPICE were kindly provided by Dr. M. Roederer Vaccine Research Center, NIH, Bethesda, MD.

Based on the results obtained with the sixteen seronegative subjects (**Figure S2**), responses were considered positive if the percentage of antigen-specific cells was 2-fold above the background and greater than 0.05% after background subtraction for at least two of the functional parameters.

### Assigning the rank order of timing of escape mutations

Sequence analysis of epitope-containing regions was performed at multiple time points over the first 87 weeks of infection. For some samples and epitopes, many single gene amplification (SGA) sequences were obtained spanning the epitope, and for others, particularly samples that were sequenced in the context of previous studies [26], only a single direct population sequence was available (see supporting information for a complete listing of epitope sequences at each time point studied). Direct sequences readily detect mixed bases at positions if they are present in roughly 25% of the population. The times at which the first evidence for selection of sequence changes in/around epitopes and the last traces of the transmitted epitope were observed were determined.

### Evaluation of the entropy of each epitopic region

To test the hypothesis that the pace of early escape may correlate with the capacity of the epitope to readily tolerate mutations, reflected in diversity of the epitope at the population level, we used Shannon entropy as a measure of epitope diversity. Shannon entropy (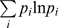 where p is the probability of the i^th^ form of the epitope), is an information theory measure of the uncertainty in a variable [55]. The Shannon entropy can be used to compare the variability in positions or regions in protein alignments [56], [57]. Here we extracted each epitope region studied from the B subtype of Los Alamos database web alignments using the database tool QuickAlign (www.hiv.lanl.gov), and used the observed frequency of each epitope variant to estimate the probability of a given variant and to calculate the entropy. The database alignments that we used contained a single randomly selected sequence per infected person, to be representative of the circulating population, and included between 500–1000 sequences, depending on what was available for a given protein. Entropy is thus reported as a measure of epitope variation at the population level; the higher the value the more variable the epitope, both in terms of the number of variant forms and the distribution of their frequencies. If the defined epitope was 9 amino acids long, we used the precise epitope fragment; if it was longer or not precisely defined within the reactive peptide, we used the 9 amino acids centered on the escape mutations observed in the subject, so each epitope region studied was exactly 9 amino acids long and thus we could compare relative entropy values between epitopes.

### Statistical analysis

A statistical test for linear trend in mean values over the six time points was computed to analyze each degree of polyfunctionality for the Gag-specific responses. A linear mixed effects (LME) model was fit to the Gag-specific longitudinal data to account for correlations amongst the measurements over time.

A generalized linear mixed model assuming a normal distribution was used to analyze the contribution of T cell responses and epitope entropy to predicting the rank order of escape across all subjects using the statistical package R (http://www.R-project.org). To determine if these variables were correlated to the within-subject rank-order of escape, we calculated the non-parametric Spearman's ρ statistic comparing either the ranks of the epitope entropy or the T cell response level with the sequential timing of escape for each subject. Spearman's ρ was calculated for each the 5 subjects with multiple epitopes studied, and the ρ's were summed. We then randomized within-subject data 100,000 times, and a recalculated the sum of the ρ's for each of the randomized data sets. This provided a Monte Carlo framework to test against the null that within-subject ranks were not correlated.

## Supporting Information

Figure S1 Gating strategy. I. The initial gates were set to include singlet events, live CD3,^+^ cells, lymphocytes, and CD4^+^ and CD8^+^ subsets. From the total CD8^+^ subset, we identified the CD45RO^−^CD27^+^ total naive subset (Naive) and excluded this from the subsequent analysis. Within the memory population, the central memory (CM) CD45RO^+^CD27^+^, effector memory (EM) CD45RO^+^CD27- and the terminal effector (TE) CD45RO^−^CD57^+^ populations were identified. Five antigen-specific populations were subsequently identified within the memory populations. II. Boolean gate combinations were also used to define cells that exhibited only one of any of the five functions ("1+"), any two of five ("2+"), and so forth, up to cells that exhibited all five functions simultaneously ("5+"). "Pies" represent the colour codes for each of the five functional families that are used in the pie charts throughout the paper.
(0.83 MB TIF)

Figure S2 Background reactivity in HIV seronegative controls. I. The dot-plots in the top row represent an example of the frequency of CD107^+^, IFN-γ^+^, IL-2^+^, MIP-1β+, and TNF-α^+^ CD8^+^ T cells identified according to the strategy illustrated in Figure S1 in samples collected from HIV-seronegative donors in the absence of antigen stimulation (unstimulated condition representing the background). In the second row, we show an example of the frequency of cells responding to stimulation with a pool of peptides representing the Gag sequence. The average frequencies (± standard deviation) of CD107^+^, IFN-γ^+^, IL-2^+^, MIP-1β^+^, and TNF-α^+^ CD8^+^ T cell populations were 0.06 (±0.04), 0.03 (±0.04), 0.05 (±0.07), 0.06 (±0.04), 0.04±0.03, respectively, for the unstimulated condition. The analysis of the responses to Gag peptide pool in the same subjects revealed average frequencies (± standard deviation) of 0.07 (±0.06), 0.06 (±0.07), 0.07 (±0.08), 0.06 (±0.06), 0.05 (±0.03) for CD107^+^, IFN-γ^+^, IL-2^+^, MIP-1β^+^, and TNF-α^+^ CD8^+^ T cell populations, respectively. II. Each figure represents the frequency of the responding cells in each of the 16 HIV seronegative donors for each functional measurement. The frequencies observed in each donor for the unstimulated and Gag peptide pool stimulated conditions are connected. In no instance was Gag-specific reactivity for each functional parameter 2-fold higher than the background and greater than 0.05 after background subtraction.
(0.51 MB TIF)

Text S1 List of transmitted/founder and escape epitope sequences utilized for the analyses.
(0.06 MB DOC)
