# Supplementary figures and images for: Relationship between Functional Profile of HIV-1 Specific CD8 T Cells and Epitope Variability with the Selection of Escape Mutants in Acute HIV-1 Infection
Source: PLoS Pathog. 2011 Feb 10;7(2):e1001273. doi: 10.1371/journal.ppat.1001273 (PMC3037354; doi:10.1371/journal.ppat.1001273)

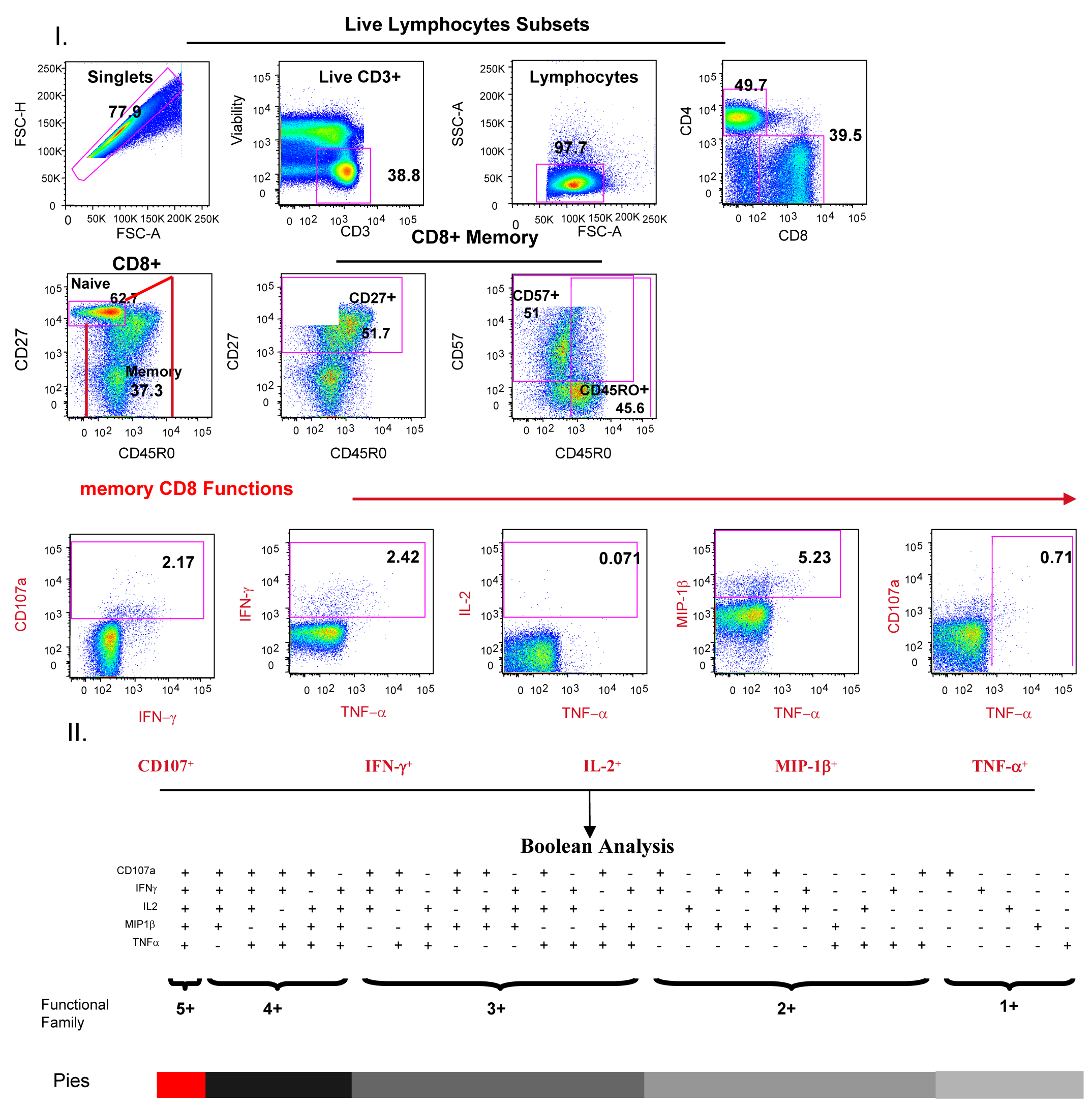

Supplement: Figure S1 — Gating strategy. I. The initial gates were set to include singlet events, live CD3,+ cells, lymphocytes, and CD4+ and CD8+ subsets. From the total CD8+ subset, we identified the CD45RO−CD27+ total naive subset (Naive) and excluded this from the subsequent analysis. Within the memory population, the central memory (CM) CD45RO+CD27+, effector memory (EM) CD45RO+CD27- and the terminal effector (TE) CD45RO−CD57+ populations were identified. Five antigen-specific populations were subsequently identified within the memory populations. II. Boolean gate combinations were also used to define cells that exhibited only one of any of the five functions ("1+"), any two of five ("2+"), and so forth, up to cells that exhibited all five functions simultaneously ("5+"). "Pies" represent the colour codes for each of the five functional families that are used in the pie charts throughout the paper. (0.83 MB TIF) [file ppat.1001273.s001.tif]

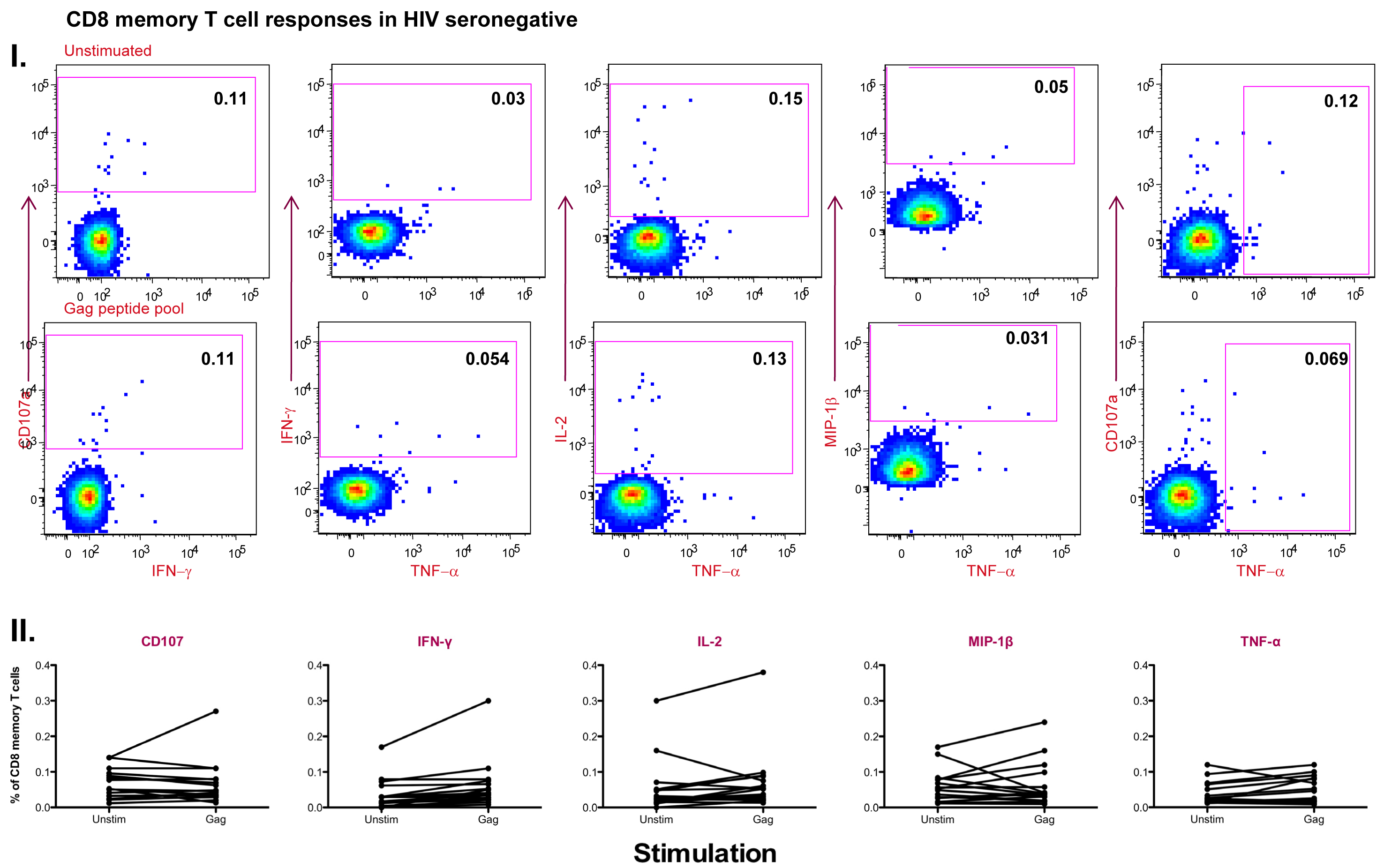

Supplement: Figure S2 — Background reactivity in HIV seronegative controls. I. The dot-plots in the top row represent an example of the frequency of CD107+, IFN-γ+, IL-2+, MIP-1β+, and TNF-α+ CD8+ T cells identified according to the strategy illustrated in Figure S1 in samples collected from HIV-seronegative donors in the absence of antigen stimulation (unstimulated condition representing the background). In the second row, we show an example of the frequency of cells responding to stimulation with a pool of peptides representing the Gag sequence. The average frequencies (± standard deviation) of CD107+, IFN-γ+, IL-2+, MIP-1β+, and TNF-α+ CD8+ T cell populations were 0.06 (±0.04), 0.03 (±0.04), 0.05 (±0.07), 0.06 (±0.04), 0.04±0.03, respectively, for the unstimulated condition. The analysis of the responses to Gag peptide pool in the same subjects revealed average frequencies (± standard deviation) of 0.07 (±0.06), 0.06 (±0.07), 0.07 (±0.08), 0.06 (±0.06), 0.05 (±0.03) for CD107+, IFN-γ+, IL-2+, MIP-1β+, and TNF-α+ CD8+ T cell populations, respectively. II. Each figure represents the frequency of the responding cells in each of the 16 HIV seronegative donors for each functional measurement. The frequencies observed in each donor for the unstimulated and Gag peptide pool stimulated conditions are connected. In no instance was Gag-specific reactivity for each functional parameter 2-fold higher than the background and greater than 0.05 after background subtraction. (0.51 MB TIF) [file ppat.1001273.s002.tif]
