# Supplementary material for: Relationship between Functional Profile of HIV-1 Specific CD8 T Cells and Epitope Variability with the Selection of Escape Mutants in Acute HIV-1 Infection
Source: PLoS Pathog. 2011 Feb 10;7(2):e1001273. doi: 10.1371/journal.ppat.1001273 (PMC3037354; doi:10.1371/journal.ppat.1001273)
Supplement: Text S1 — List of transmitted/founder and escape epitope sequences utilized for the analyses. (0.06 MB DOC) [file ppat.1001273.s003.doc]

**Table of epitope variants:** “Trans” means the transmitted virus sequence. The subject and epitope are described on the first line, followed by the epitope variation over time. Dashes mean identity with the transmitted form. The number of identical sequences is given. We put in the regions spanning or proximal to the epitope where changes occurred, and if the epitope is known or predicted, it is bold. If only the epitope is shown, it is because there was no selection outside of it. If proximal regions are shown, it is because there is some evidence for selection outside the epitope, possibly processing escape forms, and those are noted by being shaded grey. Three of the epitopes have candidates of processing escape mutations.

We are viewing single substitutions at a site as no evidence for selection, the first case of recurrent variations at the site as selection, and we have marked the first traces of selection in bold. The motivation for this strategy is that stochastic mutation occurs at a relatively high frequency in HIV throughout the genome [refs a-d], and thus a mutation observed only once in a longitudinal data set is likely to be a random event. In contrast, mutations found more than once in a given sample, or persisting mutations found in multiple time points, are clearly replicating and their recurrence evidence for selective pressure.

a) [Modeling sequence evolution in acute HIV-1 infection.](http://www.ncbi.nlm.nih.gov/pubmed/19660475)

Lee HY, Giorgi EE, Keele BF, Gaschen B, Athreya GS, Salazar-Gonzalez JF, Pham KT, Goepfert PA, Kilby JM, Saag MS, Delwart EL, Busch MP, Hahn BH, Shaw GM, Korber BT, Bhattacharya T, Perelson AS. J Theor Biol. 2009 Nov 21;261(2):341-60. Epub 2009 Aug 4.PMID: 19660475

# b) [Genetic identity, biological phenotype, and evolutionary pathways of transmitted/founder viruses in acute and early HIV-1 infection.](http://www.ncbi.nlm.nih.gov/pubmed/19487424) Salazar-Gonzalez JF, Salazar MG, Keele BF, Learn GH, Giorgi EE, Li H, Decker JM, Wang S, Baalwa J, Kraus MH, Parrish NF, Shaw KS, Guffey MB, Bar KJ, Davis KL, Ochsenbauer-Jambor C, Kappes JC, Saag MS, Cohen MS, Mulenga J, Derdeyn CA, Allen S, Hunter E, Markowitz M, Hraber P, Perelson AS, Bhattacharya T, Haynes BF, Korber BT, Hahn BH, Shaw GM.

J Exp Med. 2009 Jun 8;206(6):1273-89. Epub 2009 Jun 1.PMID: 19487424

# c) [The first T cell response to transmitted/founder virus contributes to the control of acute viremia in HIV-1 infection.](http://www.ncbi.nlm.nih.gov/pubmed/19487423) Goonetilleke N, Liu MK, Salazar-Gonzalez JF, Ferrari G, Giorgi E, Ganusov VV, Keele BF, Learn GH, Turnbull EL, Salazar MG, Weinhold KJ, Moore S; CHAVI Clinical Core B, Letvin N, Haynes BF, Cohen MS, Hraber P, Bhattacharya T, Borrow P, Perelson AS, Hahn BH, Shaw GM, Korber BT, McMichael AJ.

J Exp Med. 2009 Jun 8;206(6):1253-72. Epub 2009 Jun 1.PMID: 19487423

# d) [Identification and characterization of transmitted and early founder virus envelopes in primary HIV-1 infection.](http://www.ncbi.nlm.nih.gov/pubmed/18490657)

Keele BF, Giorgi EE, Salazar-Gonzalez JF, Decker JM, Pham KT, Salazar MG, Sun C, Grayson T, Wang S, Li H, Wei X, Jiang C, Kirchherr JL, Gao F, Anderson JA, Ping LH, Swanstrom R, Tomaras GD, Blattner WA, Goepfert PA, Kilby JM, Saag MS, Delwart EL, Busch MP, Cohen MS, Montefiori DC, Haynes BF, Gaschen B, Athreya GS, Lee HY, Wood N, Seoighe C, Perelson AS, Bhattacharya T, Korber BT, Hahn BH, Shaw GM.

Proc Natl Acad Sci U S A. 2008 May 27;105(21):7552-7. Epub 2008 May 19.PMID: 18490657

This is unevenly sampled data, and some time points include direct PCR sequences (bulk), other times a number of SGA sequences obtained from later time points. We took the data as given, and based our rate of escape classification on the available data from each individual. Previously published bulk sequences [ref 26 in the manuscript] were used in cases when samples from the earliest time points were not available for re-sequencing by SGA. Bulk sequences can reliably detect a mutation present in 20% of the sequences [ref x and y], still our capacity to detect low levels of mutation in the archival bulk sequences is diminished relative to SGA sequencing, introducing a potential bias. This bias, however, was unlikely to have had a practical impact on the escape rate classifications used in this study for the following reasons: All 4 of the epitopes classified as “non-escape”, 7 classified as early escape, and 3 classified as late escape were SGA sequenced only, so not impacted this issue. On the other hand, 5 epitopes classified as early escape and 4 classified as late escape were sequenced by bulk sequencing at early time points using data from previously published studies, and SGA sequencing at later time points, using data obtained for this study. For the 5 early escapes, the detection limitation bias introduced by bulk sequencing could not have impacted our classification, as we were in fact able to detect early escape despite having only bulk sequencing available. The 4 late escapes might have been impacted by this bias. In each of these 4 cases, however, the first time point in which escape was noted was a sample with SGA sequences available, and in each of the 4 cases, the transmitted epitope was still evident among the sampled viruses at this late time point, suggesting that escape was still in a relatively early stage at this late time point and further supporting the late escape classification. Thus, for each epitope, the escape-rate classification was based on the available data, was a reasonable inference given the data, and was supported by the observed mutational patterns.

The other data limitation faced by us, as well as by others working with human data, is sparse sampling frequencies. This supplement is provided to give full break down of the available data, to make transparent its strengths and weaknesses, and to illustrate our classification algorithm and its underlying assumptions.

Refs:

x. Biotechniques 15:120-127 (1993). Analysis of Heterologous Viral Populations by Direct DNA sequencing. T. Leitern, E Halapi, G. Scarlatti, P. Rossi, J. Albert, E.-M. Fenyo, and M. Uhlen.

y. Jordan MR, Kearney M, Palmer S, Shao W, Maldarelli F, et al. (2010) Comparison of standard PCR/cloning to single genome sequencing for analysis of HIV-1 populations. J Virol Methods 168: 114-120.

**Trans Epitope ID Pro HLA status selection-starts**

Days variants Num Freq

**CH40**

Trans **RLRDLLLIV** CH40 Gag A*0201 n-ME >412

16 --------- 9 1

45 --------- 14 1

111 --------- 9 1

181 --------- 7 1

412 --------- 13 1

Trans **KELYPLASL** CH40 Env B*4001 n-ME >412

16 --------- 13 1

45 --------- 12 1

111 --------- 8 1

181 --------- 10 0.91

181 Q-------- 1 0.09

412 --------- 11 1

Reactive peptide, eptiope unkwown:

Trans VKTIHTDNGSNFTSTTVK CH40 Pol ND n-ME >412

16 ------------------ 13 1

45 ------------------ 12 1

111 ------------------ 8 1

181 ------------------ 11 1

412 ------------------ 11 1

Possible processing escape, d111 is red as it differs with your call.

Trans qr**QIRSISERIL**s CH40 Rev A*0201 e-ME d111

16 ------------- 9 1

45 ------------- 14 1

111 ------------- 7 0.78

**111 -----------F- 1 0.11**

111 -K----------- 1 0.11

181 ------------- 6 0.86

181 -----------F- 1 0.14

412 ------------N 11 0.92

412 -----------F- 1 0.08

Trans **IEVVQRACRAILHIPRRI** CH40 Env ND l-ME d412

16 ------------------ 9 1

45 ------------------ 14 1

111 ------------------ 7 0.78

111 -----------F------ 1 0.11

111 --I--------------- 1 0.11

181 ------------------ 6 0.86

181 ----------------K- 1 0.14

412 ------------------ 10 0.83

**412 -----------C------ 1 0.08**

**412 -----------R------ 1 0.08**

Trans **AIRKAILGR** CH40 Vif A*3101 e-ME d181

16 --------- 9 1

45 --------- 14 1

111 --------- 8 0.89

111 -----L--- 1 0.11

**181 ---T----- 4 0.57**

181 ------V-- 1 0.14

181 --------- 2 0.29

412 ---E----- 9 0.75

412 --------- 3 0.25

**CH77**

Trans **QFRNKTIVF** CH77 Env Cw*0401 e-ME d14

**14 --K------ 11 0.73**

14 --------- 2 0.13

**14 --S------ 1 0.07**

14 -----A--- 1 0.07

32 --K------ 12 1

102 --K------ 4 0.8

102 --N------ 1 0.2

159 --K------ 10 0.83

159 --N------ 2 0.17

592 --K--A--- 1 0.20

592 --KD----- 1 0.20

592 --K------ 3 0.60

Trans SGEDWNKTLSHVVDKLRE CH77 Env ND e-ME d102

14 ------------------ 15 1.00

32 ------------------ 15 1.00

102 ------------------ 4 0.80

**102 ----------Y------- 1 0.20**

159 ------------------ 4 0.33

159 -------------N---- 6 0.50

159 ---------G-------- 1 0.08

159 ---------N-------- 1 0.08

592 ----------Y------- 4 0.80

592 -------------A---- 1 0.20

Trans **TSTLQEQVGW** CH77 Gag B*5701 e-ME d159

14 ---------- 17 1

32 ---------- 6 1

102 ---------- 4 1

**159 -------I-- 8 0.89**

**159 --------E- 1 0.11**

592 --N----IA- 8 1

Trans **TTTVPWNVSW** CH77 Env B*5701 l-ME d592

14 ---------- 15 1

32 ---------- 12 1

102 ---------- 5 1

159 ---------- 12 1

**592 -------T-- 3 0.6**

**592 -------S-- 2 0.4**

**CH58**

Trans **ISPRTLNAW** CH58 Gag B*5701 l-ME d350

9 --------- 7 1

45 --------- 9 1

85 --------- 9 1

154 --------- 2 1

**350 L-------- 1 0.33**

**350 X-------- 1 0.33 X is L or S**

350 --------- 1 0.33

Trans **HTQGYFPDW** CH58 Nef B*5701 n-ME >d350

9 --------- 7 1

45 --------- 9 1

85 --------- 9 1

154 --------- 1 1

350 --------- 2 1

Trans **ERYLRDQQL** CH58 Env B*1401 e-ME d9

9 --------- 5 0.71

**9 ----G---- 1 0.14**

**9 --H------ 1 0.14**

45 ----K---- 1 0.11

45 ----S---- 2 0.22

45 --------- 1 0.11

45 ----G---- 2 0.22

45 --H------ 3 0.33

85 --------- 1 0.11

85 --H------ 3 0.33

85 ----S---- 1 0.11

85 ----K---- 3 0.33

85 ----G---- 1 0.11

154 --------- 1 1

350 ----S---- 2 1

Trans **TSTLQEQIGW** CH58 Gag B*5701 e-ME d45

9 ---------- 7 1

45 ---------- 7 0.78

**45 --N------- 1 0.11**

**45 --------E- 1 0.11**

85 --------E- 6 0.67

85 --N------- 2 0.22

85 ---------- 1 0.11

154 --------E- 1 0.5

154 --N------- 1 0.5

350 --N------- 3 1

**MM33**

Trans **EEMNLPGRW** MM33 Pol e-ME d96

12 --------- Bulk

96 -D------- Bulk

201 -D-------

391 -D-------

**MM39**

Trans **QVPLRPMTYK**AA MM39 Nef l-ME d639

11 ------------ Bulk

179 ------------ Bulk

358 ------------ Bulk

**639 ------------ 1**

**639 ----------G- 8**

**639 --------F-G- 5**

821 --------F-G- 12

821 --------F--- 1

Trans KWEKI**RLRPGGKKK** MM39 Gag l-ME d639

11 -------------- Bulk

92 -------------- Bulk

358 -------------- Bulk

**639 -------------- 11**

**639 R------------- 3**

**639 T------------- 1**

**639 -----------R-- 1 d639**

821-------------- 2

821 R------------- 1

821 -----------R-R 1

821 -------------R 7

**MM42**

Trans FDSRLAFHHIARELHPEY MM42 Nef A*0201 e-ME d183

22 ------------------ Bulk

92 ------------------ Bulk

**183 ---L-------------- Bulk**

324 ---L-------------- Bulk

**MM43**

Trans **KEKGGLEGL** MM43 Nef B*0140 l-ME d552

21 --------- Bulk

101 --------- Bulk

228 --------- Bulk

368 --------- Bulk

**552 --------- 5**

**552 --------M 16**

718 --------- 2 0.2

718 --------M 8 0.8

**Trans ALQDSGLEV**NV MM43 Pol A*0201 l-ME d552

21 ----------- Bulk 1

101 ----------- Bulk 1

228 ----------- Bulk 1

368 ----------- Bulk 1

**552 ----------- 11**

**552 ------V---- 1**

**552 ------S---- 2**

**552 ----------I 12**

**552 ----------X 1**

718 ----------I 12

718 ------S---- 1

718 ------X---X 2

Trans DCFSESAIRGAILGHIVS MM43 Vif ND e-ME d101

21 ------------------ Bulk

**101 ---------R-------- Bulk**

**101 ----D----R-------- Bulk**

228 ----D----R-------- Bulk

368 ----D----R-------- Bulk

Trans **LEWRFDITL** MM43 Nef A*0201 e-ME d228

21 --------- Bulk

101 --------- Bulk

228 --------- Bulk

**228 -A------- Bulk**

**228 -Q------- Bulk**

**228 -P------- Bulk**

368 -A------- Bulk

552 -V------ 13

552 -Q------ 8

718 -V------ 9

718 -Q------ 1

Trans NYQHLWRGGIMLLWRGIM MM43 Env ND e-ME d228

21 ------------------ Bulk

101 ------------------ Bulk

**228 ----------S------- Bulk**

368 ----W------------- Bulk
